# Efficacy of dual-target iTBS on gait function and brain activation in stroke patients: a randomized, single-blinded, sham-controlled study

**DOI:** 10.3389/fneur.2025.1678850

**Published:** 2026-01-05

**Authors:** Xianbin Wang, Sijie Sun, Yan Chen, Xiaofeng Zhang, Luoyi Deng, Jing Zhang, Shuang Wu

**Affiliations:** 1 Department of Rehabilitation Medicine, Affiliated Hospital of Guizhou Medical University, Guiyang, Guizhou, China; 2 Department of Rehabilitation Medicine, Clinical Medical College, Guizhou Medical University, Guiyang, Guizhou, China

**Keywords:** stroke, lower limb motor function, iTBS, 3D gait analysis, fNIRS

## Abstract

**Objective:**

This study aimed to assess the efficacy of intermittent theta burst stimulation (iTBS) applied to the lower limb motor cortex and the contralateral cerebellar hemisphere, both individually and in combination, on walking function in stroke patients. Secondarily, the study analyzed the effects of iTBS on brain functional connectivity across the three groups.

**Methods:**

A total of 63 participants were randomly assigned to one of three groups, with 21 participants in each group: a sham stimulation group, a single-target iTBS group that stimulated the affected lower limb motor cortex, and a dual-target iTBS group that stimulated both the affected lower limb motor cortex and the contralateral cerebellum. All participants received conventional rehabilitation therapy alongside the iTBS treatment. The iTBS was administered daily at 80% of the motor evoked potential for 21 consecutive days. Outcomes were measured using the Modified Barthel Index (MBI), the Fugl-Meyer Assessment for lower extremity (FMA-LE), and the Berg Balance Scale (BBS). Additionally, three-dimensional gait analysis and functional near-infrared spectroscopy (fNIRS) were utilized to evaluate gait parameters and brain network function.

**Results:**

Following the intervention, the dual-target iTBS group exhibited significantly greater improvements in lower limb motor function (FMA-LE) and balance (BBS) compared to both the single-target and sham groups. In gait analysis, the dual-target group demonstrated superior enhancements in key parameters, including step length and swing phase, relative to the single-target group, and achieved broader gains across gait metrics when compared to the sham group. Furthermore, fNIRS analysis revealed enhanced prefrontal-lower limb motor cortex connectivity and network efficiency only in the dual-target group, which correlated with improved gait outcomes.

**Conclusion:**

Simultaneous iTBS targeting the affected lower limb motor cortex and the contralateral cerebellar motor region is more effective than single-target stimulation in enhancing lower limb motor function and balance in stroke patients. This enhanced efficacy may arise from the activation of cortical-cerebellar circuits, which improves brain network efficiency and results in better gait outcomes.

## 1 Introduction

Stroke continues to be a leading cause of long-term disability globally, with more than half of survivors facing considerable motor impairment in their lower limbs (1, 2). Hemiplegic gait often leads to an increased risk of falls, decreased community participation, and significant challenges in performing activities of daily living. As a result, restoring walking function becomes a primary objective in stroke rehabilitation. In this context, non-invasive brain stimulation techniques, such as Transcranial Magnetic Stimulation (TMS), have emerged as promising tools to enhance neuroplasticity and facilitate functional recovery following a stroke (3). iTBS, a potent and efficient TMS paradigm, can enhance cortical excitability and facilitate motor learning (4). Although research indicates that iTBS enhances swallowing, cognitive abilities, and upper limb motor function in stroke patients, its application for lower limb recovery presents distinct challenges and has been less explored (4–6). The lower limb representation in lower limb motor cortex is more deeply localized and exhibits weaker lateralized control, which may limit the efficacy of single-target, unilateral lower limb motor cortex stimulation for complex functions like gait (7–9). Accumulating evidence indicates that complex functions like gait and balance require the integration of sensory inputs across both spatial and temporal domains, along with the execution of coordinated motor patterns. These requirements likely surpass the regulatory capacity of any single cortical region (10). Consequently, stimulation confined to a single target may be insufficient to engage the full spectrum of neural networks involved in these multifaceted processes, particularly those underlying locomotion.

Gait is a complex, whole-brain activity that depends on integrated neural networks, with the cerebellum playing a critical role. Functional magnetic resonance imaging has demonstrated that the recovery of lower-limb capacity following a stroke is closely associated with the structural and functional integrity of cerebellar circuits (11). The cerebellar-thalamic-cortical (CTC) circuit is essential for coordinated locomotor control, providing online correction of movement and regulating intracortical excitability (12). Following a cortical stroke, damage to this circuit can induce cerebellar hypometabolism, which further impairs motor coordination and compromises functional recovery (13). Importantly, the cerebellum exerts a tonic inhibitory influence on the contralateral motor cortex through the dentato-thalamo-cortical pathway. As a result, applying iTBS to the contralateral cerebellum can enhance this inhibition, which in turn facilitates the ipsilateral lower limb motor cortex (14, 15). This disinhibitory mechanism suggests that iTBS applied to the cerebellum could enhance plasticity in the contralateral lower limb motor cortex. This was also confirmed in recent studies, in which it was observed that iTBS stimulation of the cerebellum using fNIRS significantly promoted activation of the contralateral motor cortex in stroke patients (16). However, a critical question remains: are the effects of cerebellar stimulation merely additive, or is there a synergistic benefit from simultaneously modulating both nodes within the CTC circuit? While single-target iTBS applied to either the lower limb motor cortex or the cerebellum has been shown to improve lower limb motor recovery by modulating one end of this network, a dual-target approach that activates the affected lower limb motor cortex while disinhibiting it through contralateral cerebellar stimulation may promote more substantial, functionally relevant, network-wide plasticity. This combined strategy could directly counteract diaschisis within the CTC circuit, potentially leading to better recovery outcomes.

Therefore, this study aims to compare the therapeutic efficacy of single-target iTBS with a dual-target iTBS protocol for lower limb motor recovery. We hypothesize that dual-target iTBS will outperform both single-target iTBS and sham stimulation in enhancing lower limb motor function, balance, and gait performance. Furthermore, we anticipate that these functional improvements will correlate with increased prefrontal-M1 connectivity and network efficiency, as assessed by fNIRS. By examining both clinical outcomes and the underlying neural mechanisms, this study will provide neurophysiological evidence in support of a more effective circuit-based rehabilitation strategy for post-stroke gait impairment.

## 2 Materials and methods

### 2.1 Participants

This was a 21-day, randomized, single-blinded, sham-controlled trial. A cohort of stroke patients hospitalized in the Department of Rehabilitation Medicine of the Affiliated Hospital of Guizhou Medical University was enrolled in this study. The following inclusion criteria were employed:

1. Patients who met the diagnostic criteria of the Chinese Stroke Association guidelines for clinical management of ischaemic cerebrovascular diseases 2023 (17), and their diagnosis was confirmed via cranial CT or MRI examination.
2. Patients with primary unilateral cerebral infarction or cerebral hemorrhage, with residual unilateral limb hemiparesis.
3. Those aged above 20 years old, with a duration of the disease of more than 1 month.
4. Patients with a Lower limb Brunnstrom’s stage on the hemiplegic side ≥ stage III, and could walk at least 10 meters independently without assistance.

The following exclusion criteria were used:

1. Those with unstable stroke condition.
2. Patients with significant cognitive impairment and cannot cooperate with the completion of the test.
3. Those with other walking abnormalities caused by other diseases, such as Parkinson’s disease, spinal cord injury, peripheral neuropathy, lower limb fracture and severe lower limb osteoarthropathy, etc.
4. Patients with a history of implantation of cardiac pacemaker, cardiac or cerebrovascular stents, bypass surgery.

The discontinuation criteria used were as follows:

1. Those with poor patient compliance, low treatment cooperation.
2. severe complications during treatment.
3. Patients who requested to withdraw from the study during treatment.

Sixty-six stroke patients were initially enrolled based on the inclusion and exclusion criteria; 3 patients withdrew during the study, resulting in a final sample of 63 hemiplegic stroke patients. The mean age of the participants was 51.99 ± 14.03 years, and the cohort included 57 males (90.5%) and 6 females (9.5%). The demographic characteristics for each group are presented in Table 1. This study was conducted in line with the ethical requirements of Guizhou Medical University Hospital (2,023,152 K) and was registered with the China Clinical Trial Center (ChiCTR2300077571). All patients signed an informed consent form.

**Table 1:** Baseline demographic and clinical characteristics of the participants.

Variables	CT	ST	DT	*χ^2^/F*	*P*
Age [ $\bar{x}\pm s$ , /Y]	50.86 ± 15.96	54.10 ± 12.45	51.00 ± 13.90	0.350	0.706
Disease duration [*M* (*P_25_*, *P_75_*), /M]	2.00(1.00, 9.50)	2.00(1.00, 4.50)	1.00(1.00, 3.00)	3.576	0.167
Sex					
Male [*n* (%)]	19(90.50)	20(95.20)	18(85.70)	1.105	0.575
Female [*n* (%)]	2(9.50)	1(4.80)	3(14.30)		
Typology					
Ischemic [*n* (%)]	11(52.40)	16(76.19)	16(76.19)	3.663	0.160
Hemorrhage [*n* (%)]	10(47.60)	5(23.81)	5(23.81)		
Hemiplegic side					
Left-hand side [*n* (%)]	8(38.10)	10(47.60)	10(47.60)	0.514	0.773
Right-hand side [*n* (%)]	13(61.90)	11(52.40)	11(52.40)		
Brunnstrom					
3 [*n* (%)]	3(14.30)	4(19.00)	3(14.30)		0.986
4 [*n* (%)]	9(42.90)	9(42.90)	10(47.60)	0.351	
5 [*n* (%)]	9(42.90)	8(38.10)	8(38.10)		

All statistical comparisons yielded *P* > 0.05, confirming no significant baseline differences between groups. CT, control group; ST, single-target iTBS group; DT, dual-target iTBS group; Y, year; M, month; χ^2^, chi-square statistic; F, F-statistic; P, *P*-value.

### 2.2 Methods

#### 2.2.1 Study groups

**Figure 1:**
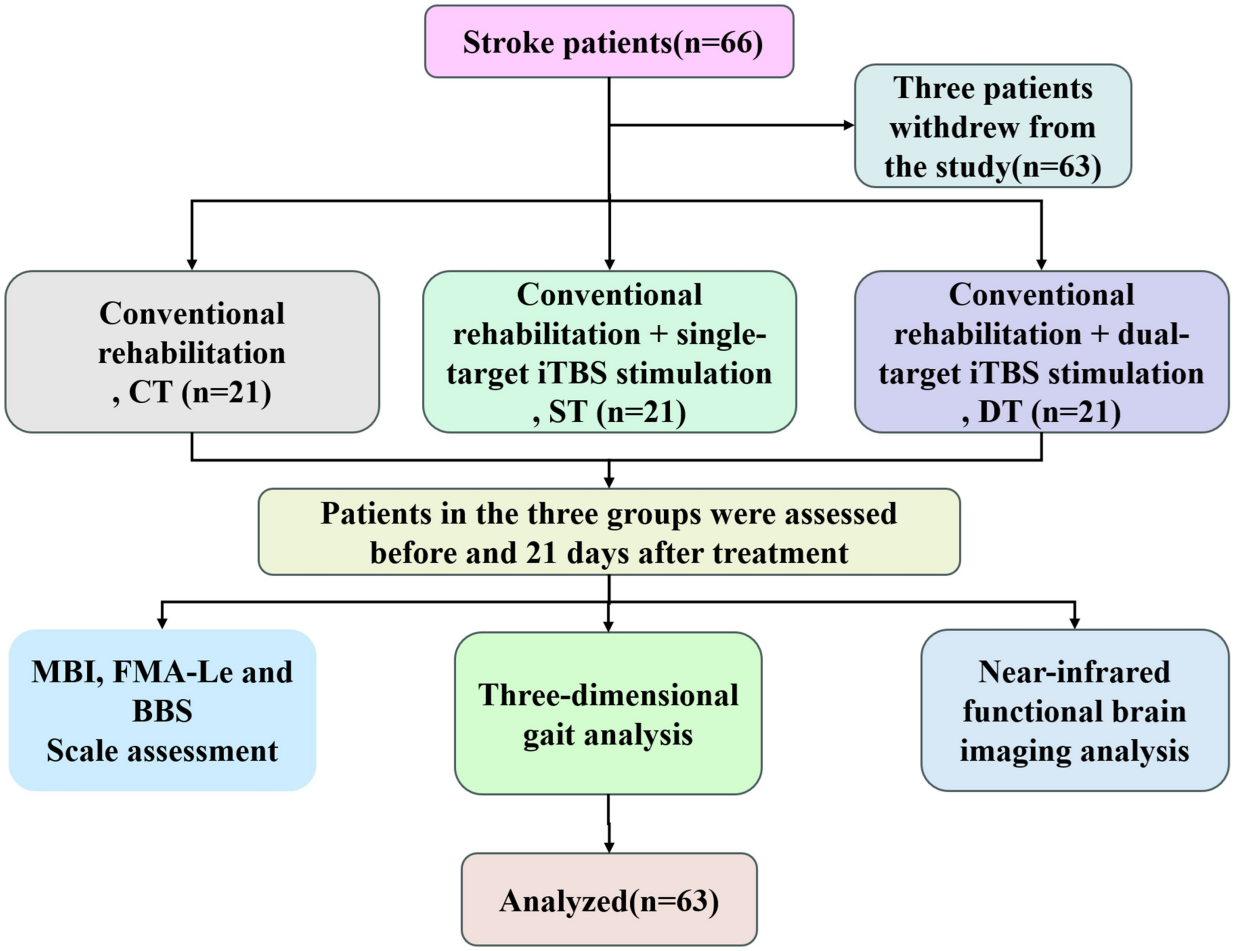
Flow chart of participant enrollment, group allocation, intervention delivery, and outcome assessment in the randomized controlled trial.

The participants (*N* = 63) were randomly assigned to three groups: a control group (*n* = 21), an iTBS single target group (*n* = 21), and an iTBS dual target group (*n* = 21). All participants received conventional rehabilitation therapy and were randomly designated to either the control group (which received placebo stimulation), the iTBS single-target group (which received iTBS intervention on the affected lower limb motor cortical area), or the iTBS double-target group (which received iTBS intervention on both the affected lower limb motor cortical area and the motor cortical area of the contralateral healthy cerebellar hemisphere) (Figure 1).

#### 2.2.2 Methods of intervention

(1) Conventional rehabilitation training methods included comprehensive training for the hemiplegic limb, endurance training, aerobic training, gait training, and balance function training. Each of these training modalities was conducted for 30 min per day over a period of 21 days. The control group used the same model of therapeutic equipment as the intervention groups, but with locked parameters. This modification ensured that the control group’s equipment produced only mild physical sensations, akin to those experienced by the intervention group, without delivering any actual stimulus intensity.
(2) iTBS treatment: A domestically produced YRD NS1000-A magnetic stimulator (Yiruide, Wuhan Yiruide Medical Equipment New Technology Co., Ltd., China). equipped with a biconical coil (maximum intensity: 1.5-6 T) was utilized. Anatomical landmarking was used for coil positioning to ensure consistency and reproducibility. Prior to the treatment, the participant was positioned comfortably on the treatment bed.
 a. iTBS single-target group: The intervention targeted the affected lower limb motor cortex, specifically the optimal stimulation site located in the median sagittal plane over the tibialis anterior muscle, where maximum evoked motor potentials (MEP) were identified via surface electromyography (EMG) recording. The stimulation intensity was set at 80% of the resting motor threshold (RMT) of the tibialis anterior muscle (RMT defined as the minimum intensity required to elicit MEPs ≥ 50 μV in 5 out of 10 consecutive trials). The parameters included an intraburst frequency of 50 Hz with 3 pulses per burst, an interburst frequency of 5 Hz with 10 bursts per train, a stimulation duration of 2 s per train, an 8-s interval between trains, and a total of 600 pulses per session. This protocol was administered for 3 min per session, once daily for 21 consecutive days.
 b. iTBS dual-target group: The two targets were stimulated consecutively in a fixed order: affected lower limb motor cortex first, followed by the contralateral cerebellar hemisphere. The lower limb motor cortex stimulation parameters were identical to those in the single-target group. For the cerebellar stimulation: The target was anatomically standardized, located 1 cm below and 3 cm lateral to the inion. The stimulation intensity was also set at 80% of the lower limb motor cortex RMT. The stimulation frequency, burst structure, and inter-train interval matched those of the lower limb motor cortex stimulation, with a total of 600 pulses for the cerebellar target. The total duration of the dual-target iTBS session was 6 min (3 min for lower limb motor cortex + 3 min for cerebellum), administered once daily for 21 consecutive days.
(3) Assessment of adverse events: All adverse events (AEs) were actively monitored for potential associations with the intervention. All AEs were thoroughly documented, and serious AEs were managed immediately and reported to the relevant ethics committee. No adverse events were observed throughout the experiment.

Adherence monitoring: Participants’ attendance was recorded daily, and treatment compliance was confirmed by checking the stimulator’s session log. Only sessions with complete pulse deliver were counted as compliant; no missed sessions were allowed, and any delays (>24 h) were documented and analyzed post-hoc (none occurred in this study).

#### 2.2.3 Scale assessment

A comprehensive scale assessment was conducted to evaluate key domains of motor recovery and functional independence among the participants. The FMA-LE was utilized to assess motor impairment in the affected lower limb. This scale measures reflexes, volitional movements, and coordination, with a maximum score of 34 points (18). The BBS was applied to assess static and dynamic balance performance. It evaluates a patient’s ability to maintain balance during a series of 14 tasks, such as standing unsupported, reaching, and turning, with a maximum score of 56 points (19). A higher score indicates improved balance control. The MBI was used to assess the patients’ performance in activities of daily living (ADL). It measures functional independence in various areas, including feeding, grooming, bathing, dressing, and mobility, with a maximum total score of 100 points (20). A higher score indicates a greater level of independence. These scales provided a comprehensive assessment of the patients’ lower limb motor function, postural control, and functional autonomy.

#### 2.2.4 Three-dimensional gait assessment

**Figure 2:**
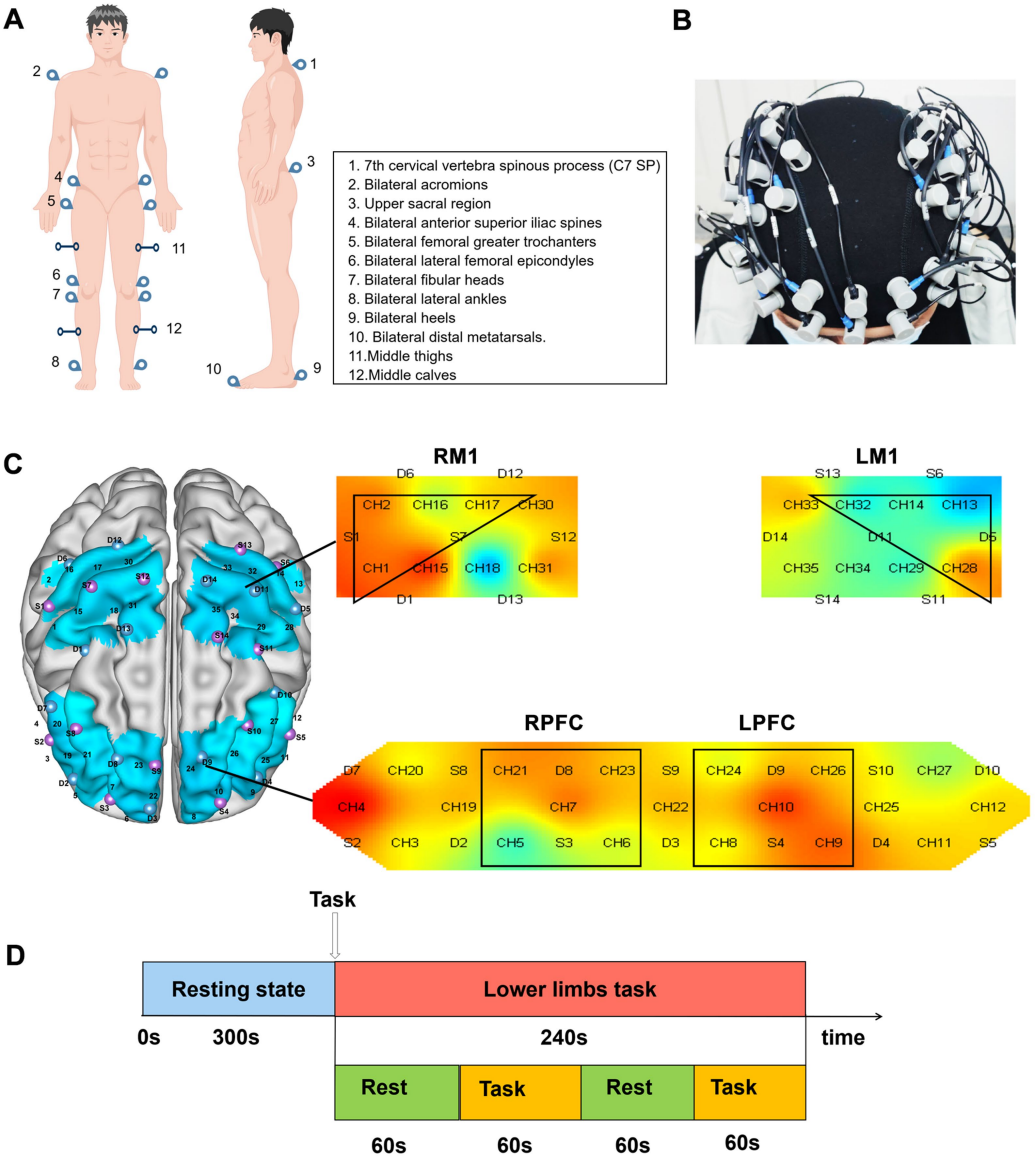
Illustration of the synchronized 3D gait and fNIRS assessment protocol. **(A,B)** Illustration of the human body indicating the key anatomical markers placed on bony landmarks for 3D gait analysis. **(C)** Layout of fNIRS optodes over the cerebral cortex, with numbered channels. The highlighted areas represent the Regions of Interest (ROI) selected for analysis. (M1) Configuration of fNIRS channels corresponding to specific ROIs over the motor cortical areas, respectively. (PFC) Configuration of fNIRS channels covering the prefrontal cortex (PFC). **(D)** The experimental timeline, comprising an initial 5-min resting-state period, followed by a lower limb motor task block. The task block employed an alternating paradigm, with 60-s task periods interspersed with 60-s rest periods.

In this study, participants completed walking tasks while wearing a portable fNIRS device, and a 3D gait analysis system (SMART-DX 700, BTS, Italy) was used simultaneously to collect gait parameters. A quiet examination room, free from magnetic field interference, was prepared, and a 5-meter walkway was established. The 3D gait analysis system was calibrated accordingly. Participants wore tight-fitting clothing, and reflective markers were strategically placed on various anatomical sites, including the 7th cervical vertebra spinous process, bilateral shoulder crests, upper sacral region, bilateral anterior superior iliac spines, bilateral femoral greater trochanters, bilateral lateral femoral epicondyles, bilateral fibular heads, bilateral lateral ankles, bilateral heels, and bilateral distal metatarsals. Additionally, four reflective markers were secured with elastic bands on the middle thighs and calves, resulting in a total of 22 markers (Figure 2A). The primary spatial and temporal parameters measured included temporal aspects such as swing phase, single-support phase, and stride frequency, alongside spatial parameters like stride length and stride width, and spatiotemporal parameters including stride speed. Kinematic parameters were also assessed, which encompassed the maximum angles of hip, knee, and ankle flexion, as well as the foot deviation angle.

#### 2.2.5 Near infrared brain function assessment

In this experiment, NirSmart-6000A equipment (Danyang Huichuang Medical Equipment Co., Ltd., China) was used to continuously measure and record the concentration changes of brain oxygenated hemoglobin (HbO) and deoxy hemoglobin (HbR) during the task. The system consists of a near-infrared light source (light emitting diodes, LED) and an avalanche photodiode (APD) as detectors, with wavelengths of 730 nm and 850 nm, respectively, and a sampling rate of 11 Hz. The experiment uses 14 light sources and14 detectors to form 35 effective channels, the average distance between the source and the detector is 3 cm (range 2.7–3.3 cm), with reference to the international 10/20 system for positioning. The spatial locations of sources and detectors were measured by an electromagnetic 3D digitizer device (Patriot, Polhemus, USA) placed on the head of the subject, with acquired coordinates that are converted into coordinates in line with the Montreal Neurological Institute and Hospital (MNI). These coordinates are further projected to the MNI standard brain template using the spatial registration approach in NirSpace (Danyang Huichuang Medical Equipment Co., Ltd., China). A flexible headgear holder was used to reduce signal noise between the emitter and scalp. During the experiment, the excessive light was controlled for better data collection. A brain location map with its distribution of channels is shown in Figures 2B,C (21).

#### 2.2.6 Near infrared data processing and analysis

The NIRspark (v1.8, Huichuang, China) based on Matlab (v2021a, Natick, USA) was used to analyze the experimental data collected by the fNIRS system. The data processing was performed in the following steps:

1. Setting the signal standard deviation threshold to 6 and peak threshold to 0.5.
2. Removing motion artifacts via spline interpolation.
3. Filtering physiological noise (heartbeat, respiration, and Mayer waves) using a 0.01–0.1 Hz band-pass filter.
4. Converting filtered optical density data to oxyhemoglobin (HbO) concentration using the modified Beer–Lambert law with a differential pathlength factor (DPF) of 6 (22).
5. For the task-state analysis, a block design was adopted. Each task block was defined as a 60-s period of lower limb motor task, consistent with the paradigm illustrated in Figure 2D. The baseline period was defined as the 10 s immediately preceding the task onset (−10 to 0 s). The mean HbO concentration during the task block was calculated and compared against the mean baseline value to serve as the index of activation for the corresponding brain regions.
6. After pre-processing, the resting state data could be further analysed. Due to the different lesion sites of the patients, the data of the patients with the right-sided lesion were flipped, and the left side was taken as the lesion side after flipping. For functional connectivity analysis, Pearson’s correlation coefficients were calculated between the preprocessed HbO time series from all pairs of regions of interest (ROIs) during the resting state, resulting in a correlation matrix for each participant. For analyzing topological properties of complex brain networks, the resting-state functional connectivity matrices were first converted into binary adjacency matrices. A proportional threshold of 30% was applied, meaning only the top 30% of the strongest connections were retained for subsequent graph theory analysis. A random network was generated—matching the actual network in node count, edge count, and degree distribution—to verify the reliability of the real network. The clustering coefficient (Cp) was used to quantify local connectivity and community structure of the brain network based on resting-state data. This coefficient measures the extent of functional segregation in the brain’s functional network, reflecting the network’s capacity for specialized information processing (23, 24).

### 2.3 Statistical analysis

The measurement data collected in this study were analyzed using SPSS version 26.0. The chi-square test was used to compare categorical data, while continuous variables were reported as mean ± standard deviation. The normality of all continuous data was assessed using the Shapiro–Wilk test. The mean values for Scale assessment, gait parameters (including spatio-temporal and kinematic parameters), NIR resting-state functional connectivity parameters, and task-state HbO conformed to normality. For within-group comparisons of these normally distributed indicators, paired-sample t-tests were utilized. For between-group comparisons involving three groups, one-way analysis of variance (One-way ANOVA) was first applied to test for overall differences among the groups. If the overall difference was statistically significant (*p* < 0.05), Bonferroni correction was used for multiple pairwise comparisons to control for type I error due to repeated comparisons. In contrast, for between-group comparisons involving only two groups, independent-sample t-tests were employed. Conversely, NIR clustering coefficients, shortest path lengths, global efficiencies, and local efficiencies did not follow a normal distribution. For within-group comparisons of these non-normally distributed indicators, Wilcoxon tests were applied; for between-group comparisons involving three groups, Kruskal-Wallis tests were used to assess overall differences among the groups. If the overall difference was statistically significant (*p* < 0.05), Dunn correction was further used for multiple pairwise comparisons to control the type I error. For between-group comparisons involving only two groups, Mann–Whitney U tests were used. Effect sizes are reported alongside *p*-values to indicate practical significance: Cohen’s d for t-tests (parametric), partial eta-squared (ηp^2^) for one-way ANOVA (parametric), and Spearman’s r for non-parametric tests (Wilcoxon signed-rank test, Kruskal-Wallis test, Mann–Whitney U test). A *p* < 0.05 was considered statistically significant.

## 3 Results

### 3.1 Scale assessment

**Figure 3:**
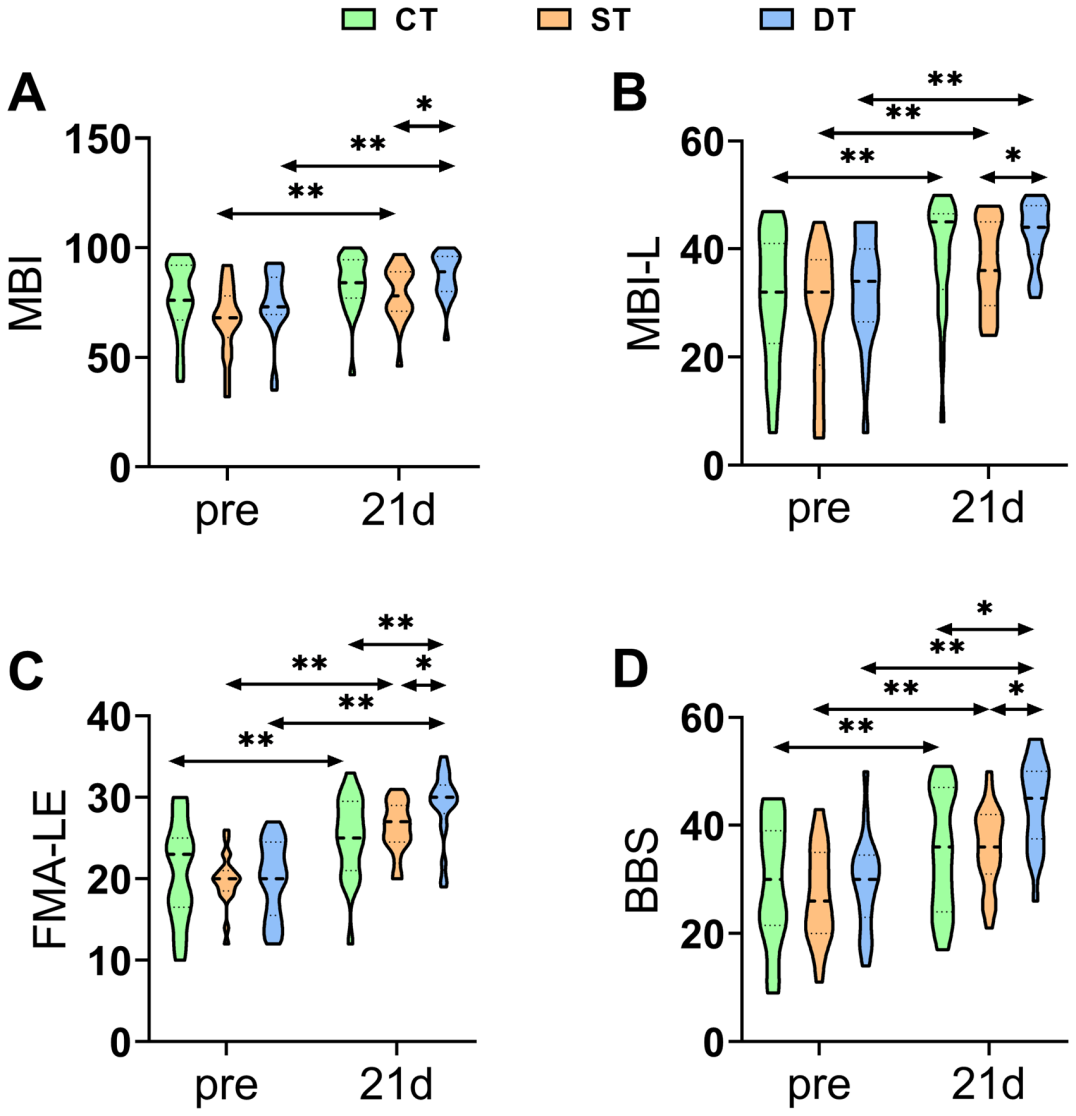
Distribution of functional outcome scores across groups before and after iTBS intervention. **(A)** Modified Barthel index (MBI), **(B)** Lower extremity MBI (MBI-L), **(C)** Fugl-Meyer Assessment for Lower Extremity (FMA-LE), and **(D)** Berg Balance Scale (BBS) at pre and after 21 days of intervention. Statistical annotations: *p < 0.05, **p < 0.01 (paired-sample *t*-test for within-group comparisons; one-way ANOVA with Bonferroni *post hoc* test for between-group comparisons).

To evaluate the improvement in patient function following iTBS, we analyzed scores from the MBI, MBI-L, FMA-LE, and BBS. A one-way ANOVA was first conducted to confirm baseline comparability, which indicated no statistically significant differences among the three groups in pre-treatment MBI, MBI-L, FMA-LE, or BBS scores (*p* > 0.05). After the 21-day intervention period, paired-sample t-tests were conducted to evaluate the effects of the treatment. The results indicated statistically significant improvements from baseline to post-treatment in MBI, MBI-L, FMA-LE, and BBS scores across all three groups (*p* < 0.05). Additionally, a one-way ANOVA was utilized to investigate the differences between groups after the intervention. Significant main effects were observed among the three groups in post-intervention scores for MBI (*F*(2, 60) = 3.437, *p* = 0.039, η^2^ = 0.103), MBI-L (F(2, 60) = 3.441, *p* = 0.038, η^2^ = 0.103), FMA-LE (F(2, 60) = 6.774, *p* = 0.002, η^2^ = 0.184), and BBS (F(2, 60) = 8.086, *p* = 0.001, η^2^ = 0.212). Post-hoc analyses with Bonferroni correction revealed that both the single-target and dual-target stimulation groups showed significantly higher scores in MBI, MBI-L, FMA-LE, and BBS compared to the control group (*p* < 0.05). The post-hoc tests revealed that the dual-target stimulation group showed significantly greater improvement compared to the single-target stimulation group. These findings collectively suggest that iTBS is effective in promoting functional recovery, with dual-target stimulation leading to better outcomes than single-target stimulation in enhancing activities of daily living, motor function, and balance (Figure 3).

### 3.2 Three-dimensional gait analysis

#### 3.2.1 Comparison of spatiotemporal parameters

**Figure 4:**
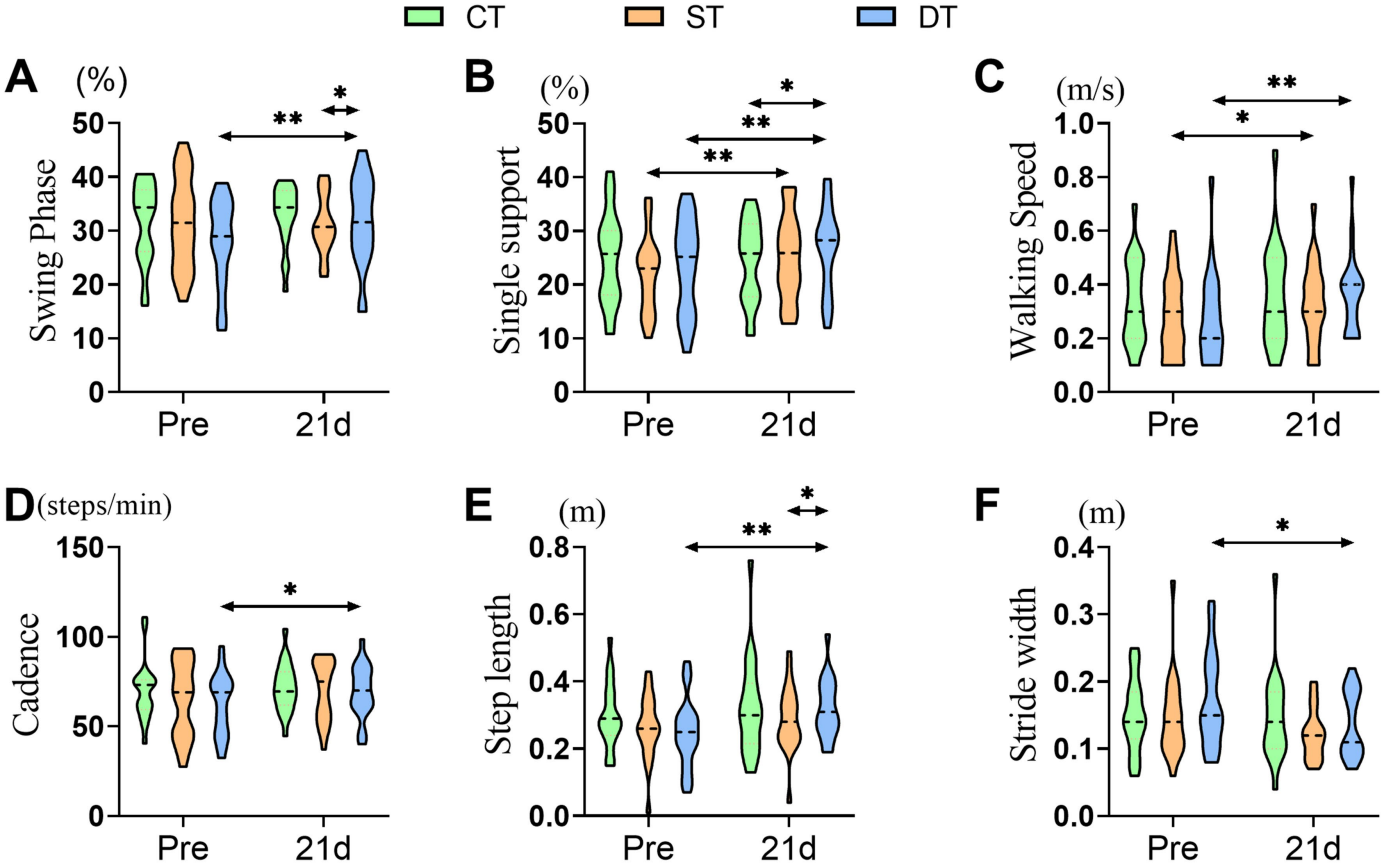
Distribution of gait spatiotemporal parameters across groups before and after iTBS intervention. **(A)** Swing phase (%), **(B)** Single support (%), **(C)** Walking speed (m/s), **(D)** Cadence (steps/min), **(E)** Step length (m), and **(F)** Stride width (m) at pre and after 21 days of intervention. Statistical annotations: *p < 0.05, **p < 0.01 (paired-sample *t*-test for within-group comparisons; one-way ANOVA with Bonferroni post hoc test for between-group comparisons).

To assess the impact of iTBS on walking ability, we initially examined spatiotemporal gait parameters. A one-way ANOVA revealed no statistically significant differences in these parameters at baseline among the three groups (*p* > 0.05), indicating that the groups were comparable prior to the intervention. Following the 21-day intervention period, we conducted paired-sample t-tests to evaluate changes within each group. In the single-target stimulation group, significant improvements were observed in the single support phase (t (20) = −4.101, *p* = 0.001) and gait speed (t (20) = −2.351, *p* = 0.029) compared to pre-treatment values. The dual-target stimulation group exhibited the most extensive improvements, with significant post-treatment increases in swing phase (t(20) = −3.093, *p* = 0.006), single support phase (t(20) = −2.899, *p* = 0.009), gait speed (t(20) = −4.954, *p* < 0.001), cadence (t(20) = −4.241, *p* < 0.001), step length (t(20) = −4.524, p < 0.001), and step width (t(20) = 2.434, *p* = 0.024). One-way ANOVA of post-intervention data revealed significant main effects among the three groups in swing phase (*F* (2, 60) = 7.389, *p* = 0.025, ηp^2^ = 0.111), single support phase (F (2, 60) = 11.301, *p* = 0.004, ηp^2^ = 0.132), and step length (F (2, 60) = 7.365, p = 0.025, ηp^2^ = 0.107). Post-hoc analyses revealed that the single-target group had a significant advantage over the control group only during the single support phase (*p* < 0.05). In contrast, the dual-target group outperformed the control group in both the single support phase and step length (*p* < 0.05). These findings suggest that dual-target iTBS is more effective in enhancing gait symmetry, as evidenced by improvements in the single support and swing phases, as well as in walking efficiency, indicated by increased step length (Figure 4).

#### 3.2.2 Comparison of kinematic parameters

**Figure 5:**
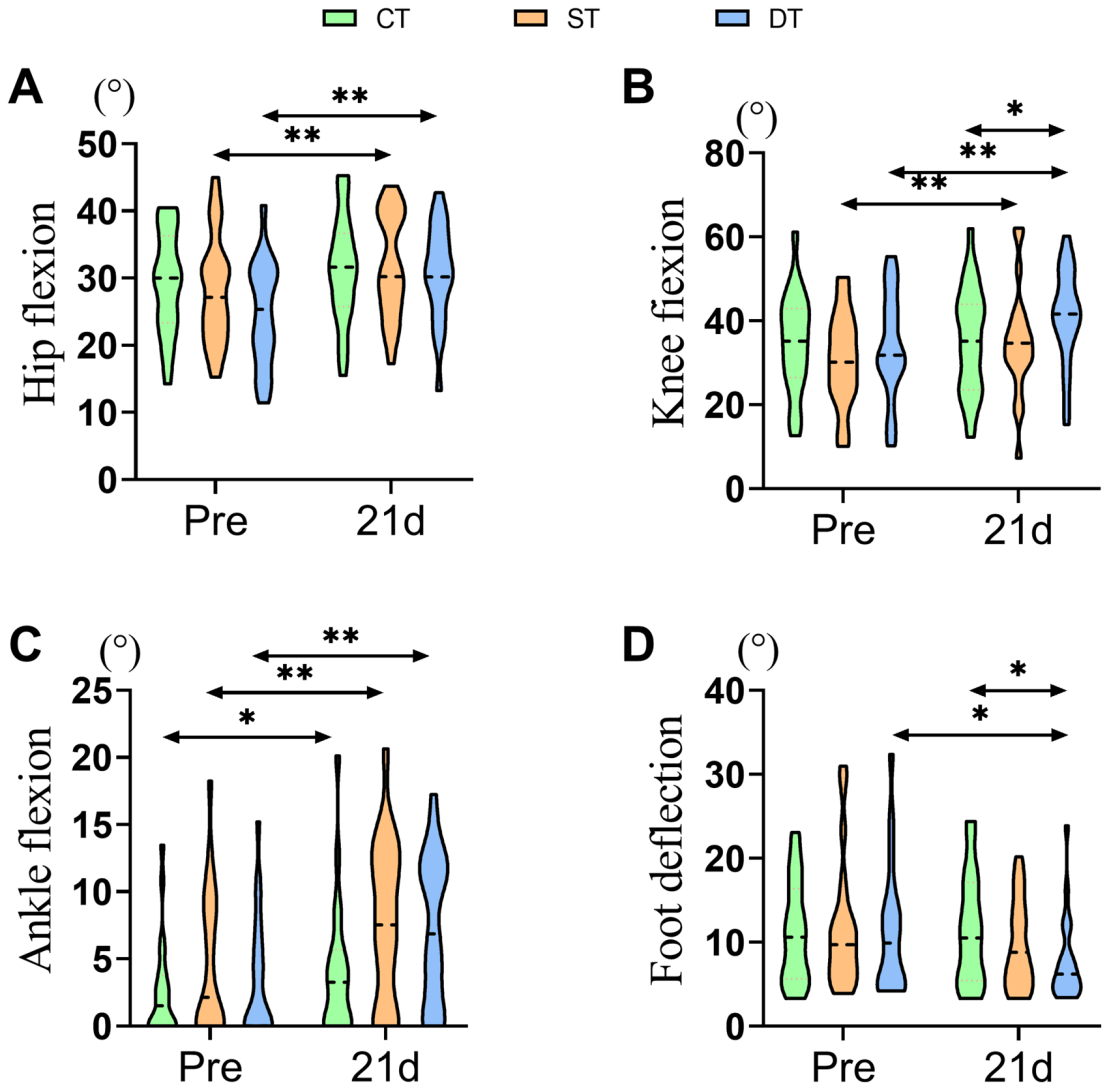
Distribution of lower extremity kinematic parameters across groups before and after iTBS intervention. **(A)** Hip Flexion (°), **(B)** Knee Flexion (°), **(C)** Ankle Flexion (°), and **(D)** Foot Deflection (°) at pre and after 21 days of intervention. Statistical annotations: *p < 0.05, **p < 0.01 (paired-sample *t*-test for within-group comparisons; one-way ANOVA with Bonferroni *post hoc* test for between-group comparisons).

To determine the impact of iTBS on gait quality and joint coordination, we further analyzed kinematic angles of the hip, knee, ankle, and foot. After the 21-day intervention period, paired-sample t-tests were conducted to evaluate within-group changes. The control group showed significant post-treatment improvements only in hip flexion (t (20) = −3.631, *p* = 0.002) and ankle dorsiflexion (t (20) = −2.702, *p* = 0.014). The single-target stimulation group exhibited significant improvements in hip flexion (t (20) = −3.416, *p* = 0.003), knee flexion (t (20) = −3.521, *p* = 0.002), and ankle dorsiflexion (t (20) = −3.943, *p* = 0.001). In contrast, the dual-target stimulation group demonstrated the most comprehensive kinematic improvements, with significant enhancements in hip flexion (t(20) = −5.574, *p* < 0.001), knee flexion (t(20) = −5.679, p < 0.001), ankle dorsiflexion (t(20) = −4.413, *p* < 0.001), as well as a significant reduction in foot progression angle (t(20) = 2.240, *p* = 0.037), indicating improved gait stability. One-way ANOVA of post-intervention data revealed significant main effects among the three groups in hip flexion (*F* (2, 60) = 7.767, *p* = 0.021, ηp^2^ = 0.147), knee flexion (F (2, 60) = 10.042, *p* = 0.007, ηp^2^ = 0.151), and foot progression angle (F (2, 60) = 6.320, *p* = 0.042, ηp^2^ = 0.067). Post-hoc analyses indicated that compared to the control group, the dual-target stimulation group showed significantly greater improvements in hip flexion, knee flexion, and foot progression angle (*p* < 0.05). However, no significant between-group main effect was observed for ankle dorsiflexion (F (2, 60) = 4.652, *p* = 0.098). Notably, although the dual-target group exhibited numerically greater improvements than the single-target group across multiple parameters, direct comparisons between the two stimulation groups did not reach statistical significance for any kinematic measure (*p* > 0.05). In summary, these results suggest that iTBS, particularly the dual-target protocol, effectively promotes the recovery of multi-joint coordinated movements in the lower extremities, thereby facilitating a more normalized gait pattern in post-stroke patients (Figure 5).

### 3.3 fNIRS

#### 3.3.1 Comparison of the mean HbO values for near-infrared brain function task states

**Figure 6:**
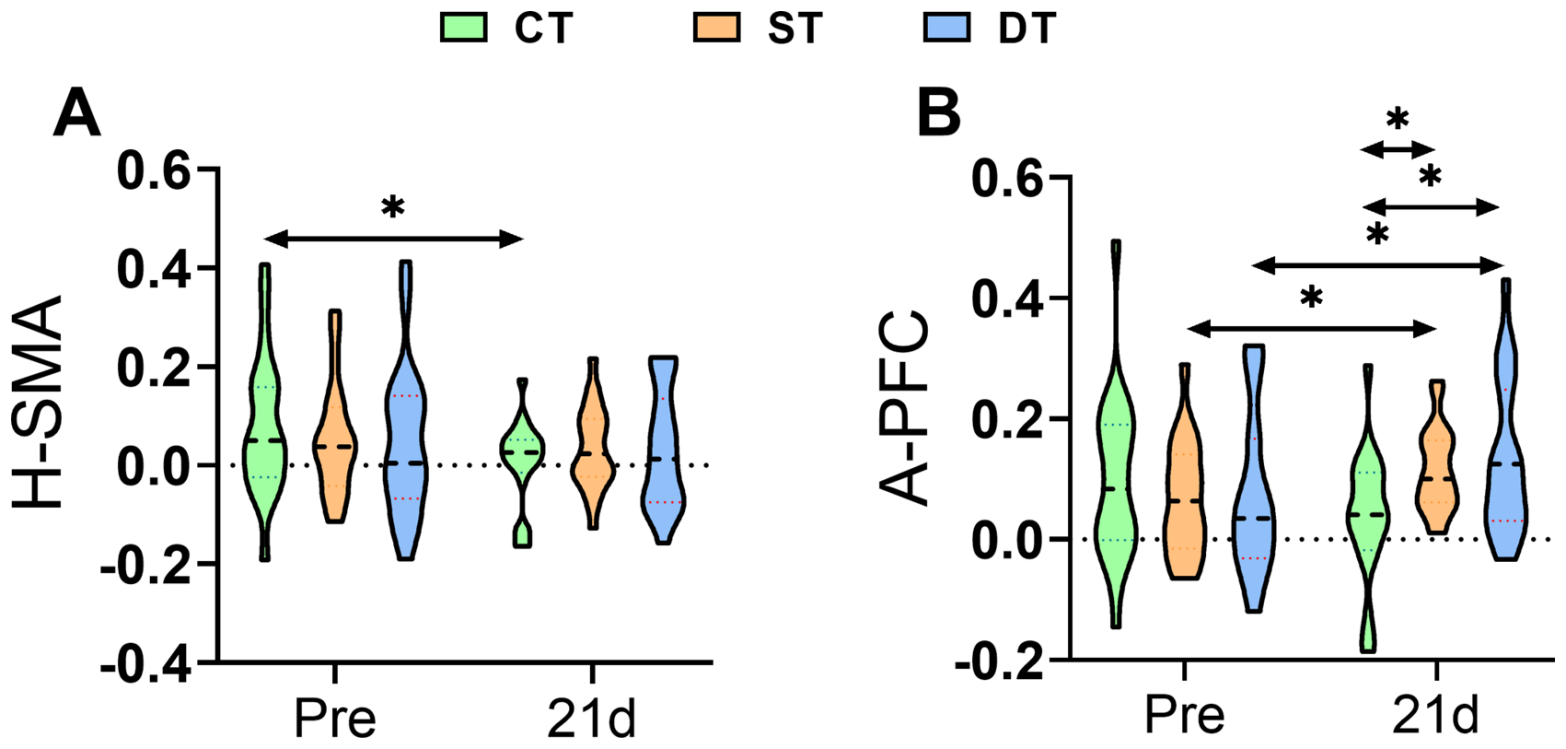
Distribution of task-evoked HbO concentration in specific ROIs across groups before and after iTBS intervention. **(A)** H-SMA (healthy supplementary motor area) and **(B)** A-PFC (affected prefrontal cortex) at Pre and after 21 days of intervention. Statistical annotation: *p < 0.05 (paired-sample *t*-test for within-group comparisons; one-way ANOVA with Bonferroni *post hoc* test for between-group comparisons).

To assess how iTBS modulates task-evoked cortical activity, we compared changes in the mean concentration of oxygenated hemoglobin (HbO) within specific regions of interest (ROIs) before and after the intervention across groups. Within-group analysis revealed that the control group showed a significant decrease in HbO concentration in the unaffected supplementary motor area after intervention (t (20) = 2.182, *p* = 0.041). In contrast, both iTBS intervention groups exhibited enhanced activation in the affected prefrontal cortex—a region associated with motor learning and higher cognitive functions. Specifically, significant increases in HbO concentration were observed in the single-target group (t (20) = −2.151, *p* = 0.044) and the dual-target group (t (20) = −2.685, *p* = 0.014) compared to their pre-treatment levels. Between-group comparisons further confirmed the specific modulatory effect of iTBS on activation of the affected prefrontal cortex. One-way ANOVA of post-intervention data indicated a significant main effect among the three groups in HbO concentration in this region (*F* (2, 60) = 7.220, *p* = 0.002, ηp^2^ = 0.194). Post-hoc tests with Bonferroni correction demonstrated that both the single-target (*p* = 0.018) and dual-target (*p* = 0.008) groups had significantly higher HbO concentrations in the affected prefrontal cortex compared to the control group. These findings suggest that iTBS intervention, whether applied in a single- or dual-target manner, effectively enhances neural activity in the affected prefrontal cortex during task performance in post-stroke patients, which may functionally compensate for impairments in the primary motor region (Figure 6).

#### 3.3.2 Comparison of resting-state ROI connectivity for near-infrared brain functions

**Figure 7:**
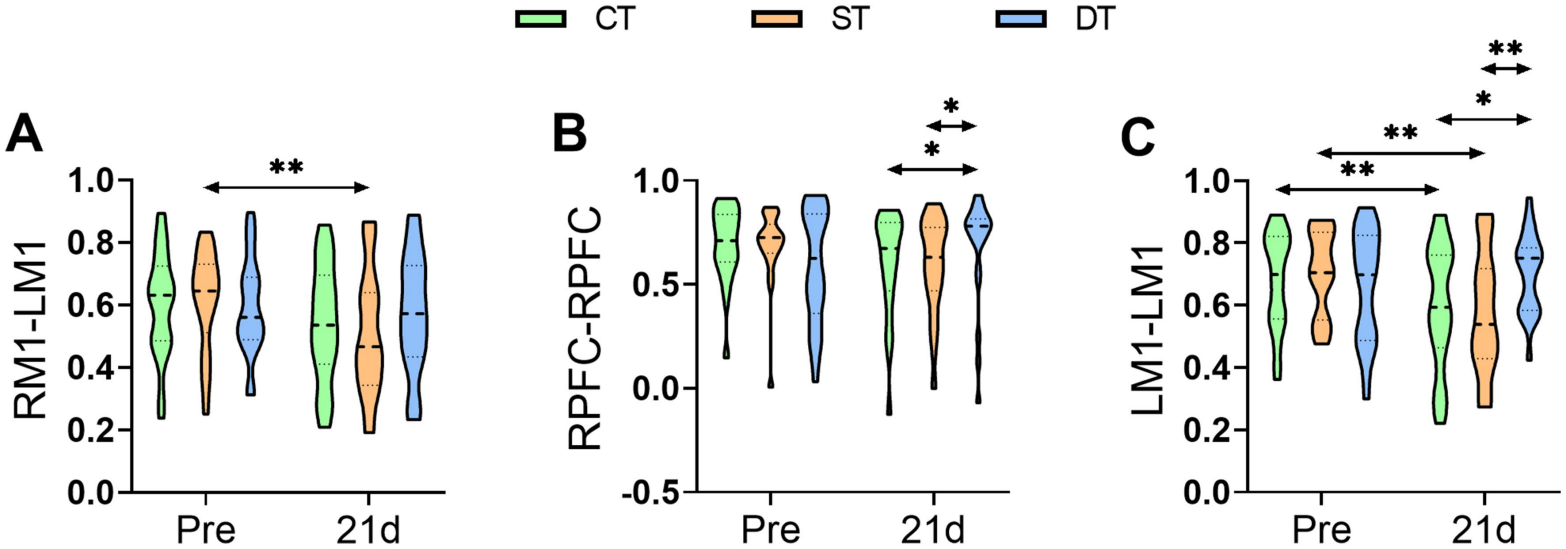
Distribution of functional connectivity strength across groups before and after iTBS Intervention assessed by fNIRS. **(A)** RM1-LM1, **(B)** RPFC-RPFC, and **(C)** LM1-LM1 at Pre and after 21 days of intervention. Statistical annotations: *p < 0.05, **p < 0.01 (paired-sample *t*-test for within-group comparisons; one-way ANOVA with Bonferroni *post hoc* test for between-group comparisons).

To investigate the impact of iTBS on functional reorganization within cortical networks, functional connectivity between specific regions of interest (ROIs) was assessed using fNIRS. Within-group analyses revealed that the control group exhibited a significant decrease in local connectivity within the affected M1 region after the intervention (t (20) = 2.446, *p* < 0.01). Similarly, the single-target stimulation group showed a weakening of functional connectivity, with significant reductions observed both in local connectivity within the affected M1 and in interhemispheric connectivity between the unaffected and affected M1 regions (*p* < 0.01). In contrast, the dual-target stimulation group demonstrated no significant changes in any of the measured functional connections from pre- to post-intervention (*p* > 0.05), suggesting a potential network-stabilizing effect unique to this form of stimulation. Between-group comparisons via one-way ANOVA of post-intervention data identified a significant main effect for local connectivity within the unaffected prefrontal cortex (*F* (2, 60) = 3.315, *p* = 0.043, ηp^2^ = 0.100). Post-hoc tests indicated that the dual-target group had significantly stronger connectivity in this region compared to the control group (*p* < 0.05). More importantly, a significant main effect was also found for local connectivity within the affected M1 region (F (2, 60) = 4.813, *p* = 0.012, ηp^2^ = 0.138). Subsequent comparisons showed that the dual-target group not only exhibited significantly greater connectivity than the control group (*p* = 0.027) but also demonstrated significantly higher connectivity strength than the single-target group (*p* = 0.004). In summary, these findings indicate that conventional therapy and single-target iTBS were unable to counteract the interhemispheric inhibition resulting from unilateral brain injury. Conversely, dual-target iTBS specifically enhanced functional connectivity in key brain regions associated with higher-order motor control and planning (unaffected prefrontal cortex) and motor execution (affected M1), which may represent the neural mechanism underlying its superior clinical efficacy (Figure 7).

#### 3.3.3 Topological property analysis of brain networks based on graph theory

**Figure 8:**
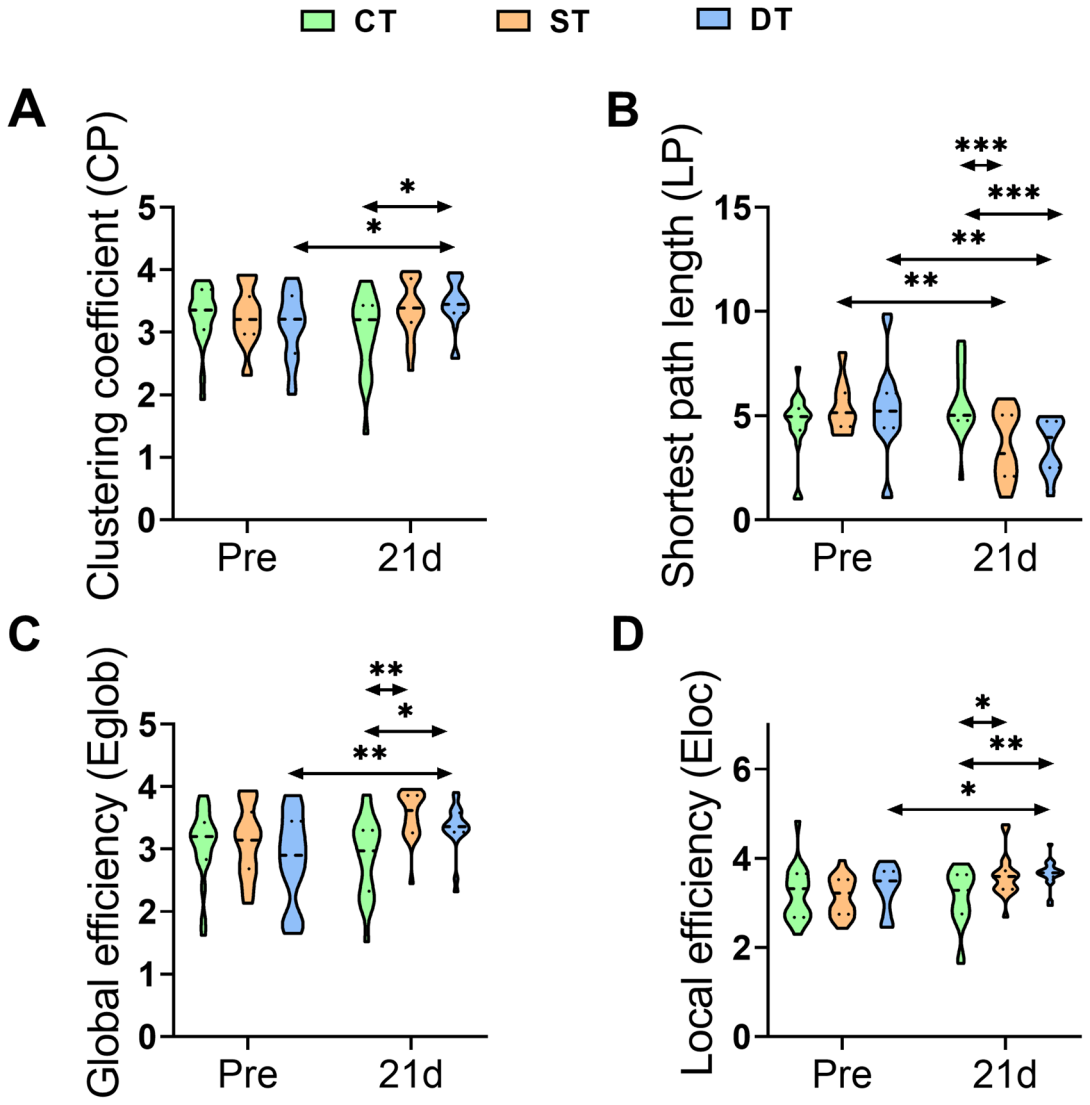
Distribution of brain functional network topological properties across groups before and after iTBS intervention. **(A)** Clustering coefficient (Cp), **(B)** Shortest path length (LP), **(C)** Global efficiency (Eglob), and **(D)** Local efficiency (Eloc). Statistical annotations: *p < 0.05, **p < 0.01, ***p < 0.001 (Wilcoxon signed-rank test for within-group comparisons; Kruskal-Wallis test with Dunn post hoc test for between-group comparisons).

To explore the effect of iTBS on the organizational efficiency of brain functional networks from a global perspective, we analyzed topological properties based on graph theory. We focused on characteristic path length and global efficiency, as well as clustering coefficient and local efficiency. Within-group comparisons using the Wilcoxon signed-rank test revealed that the control group showed no significant changes in any network properties after the intervention (*p* > 0.05). The single-target stimulation group demonstrated enhanced global network integration, with significantly shortened characteristic path length (Z = −2.868, *p* = 0.004) and increased global efficiency (Z = −3.041, *p* = 0.002). In contrast, the dual-target stimulation group exhibited the most comprehensive network optimization, showing not only significantly reduced characteristic path length (Z = −3.250, *p* = 0.001) and increased global efficiency (Z = −3.111, *p* = 0.002), but also enhanced clustering coefficient (Z = −3.007, *p* = 0.003) and local efficiency (Z = −2.103, *p* = 0.035), indicating improved local specialized processing. Between-group comparisons further confirmed the effectiveness of iTBS in reshaping brain network topology. Kruskal-Wallis tests of post-intervention data revealed significant group differences in clustering coefficient (H (2) = 8.202, *p* = 0.017, r = 0.33), characteristic path length (H (2) = 17.496, *p* < 0.001, r = 0.50), and global efficiency (H (2) = 15.417, *p* < 0.001, r = 0.48). Post-hoc Dunn tests demonstrated that compared to the control group, the single-target group showed a significantly higher clustering coefficient (*p* = 0.044, r = 0.28) and shorter characteristic path length (*p* = 0.001, r = 0.42). The dual-target group exhibited superior overall network properties, with significantly higher clustering coefficient (*p* = 0.006, r = 0.38) and global efficiency (*p* = 0.014, r = 0.35), as well as significantly shorter characteristic path length (*p* < 0.001, r = 0.52) compared to the control group. These findings indicate that single-target stimulation primarily enhances local clustering properties, whereas dual-target stimulation improves both global integration and local specialized processing capabilities in brain networks (Figure 8).

### 3.4 Correlation analysis results

**Figure 9:**
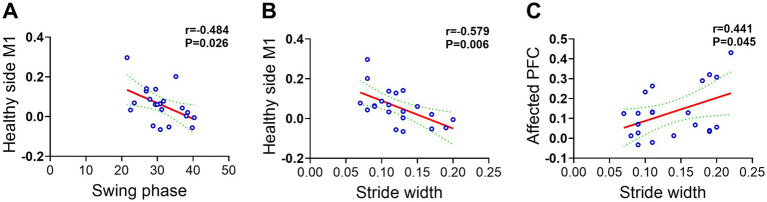
Presents scatter plots that illustrate the correlation between gait parameters and the mean value of brain region activation following single-target and dual-target iTBS treatment. **(A)** The scatter plot shows the relationship between the mean HbO value in the M1 region of the healthy side and the swing phase in the single-target group. **(B)** Scatter plot indicating the correlation between the mean HbO value in the M1 region of the healthy side and the step width in the single-target group. **(C)** The scatter plot illustrates the correlation between the mean HbO value in the PFC region of the affected side and the step width in the dual-target group.

To examine the association between post-intervention gait parameters and activation levels in specific brain regions, and to explore the potential brain-behavior mechanisms underlying iTBS therapeutic effects, Pearson correlation analyses were conducted. In the single-target stimulation group, the mean HbO concentration in the contralesional M1 region showed significant negative correlations with both the swing phase (r = −0.484, *p* = 0.026) and step width (r = −0.579, *p* = 0.006). These findings suggest that under single-target stimulation, the contralesional motor cortex may regulate both temporal swing characteristics and postural stability of gait through distinct neural pathways (Figures 9A,B). Notably, in the dual-target stimulation group, correlation analysis revealed a significant positive correlation between the mean HbO concentration in the ipsilesional prefrontal cortex (PFC) and step width (r = 0.441, *p* = 0.045) (Figure 9C). This finding suggests that the therapeutic effects of dual-target iTBS may be closely associated with higher-order motor control and postural regulation functions mediated by the ipsilesional prefrontal cortex. In summary, correlation analyses reveal distinct neural mechanisms underlying single-target and dual-target iTBS in improving gait. The effects of single-target iTBS are primarily associated with activation of the contralesional motor cortex. In contrast, the superiority of dual-target iTBS seems to involve the recruitment of the ipsilesional prefrontal cortex, a region associated with higher-order motor control. This difference underscores the neural basis for the more comprehensive therapeutic benefits of dual-target iTBS. It is noteworthy that, for both groups, no other significant correlations were found between the remaining gait parameters and the other assessed brain regions (the SMA and the contralesional PFC), with all *p* > 0.05 (Supplementary Tables S1, S2).

## 4 Discussion

This study demonstrates that dual-target iTBS, applied to the affected lower-limb motor cortex and contralateral cerebellum, yields significantly superior outcomes in improving lower extremity motor function, balance, and gait in stroke patients compared to both single-target iTBS and conventional rehabilitation. The therapeutic advantage was evident in clinical scales such as FMA-LE and BBS. More importantly, it was systematically validated through 3D gait analysis and fNIRS, which demonstrated the synergistic effects of dual-target stimulation at both behavioral and neural levels. These effects were closely associated with enhanced connectivity between the PFC and M1, optimized global and local brain network efficiency, and the correction of abnormal gait patterns—results that were not achieved with single-target stimulation.

The behavioral improvements observed in the dual-target iTBS group were directly supported by fNIRS-derived neural evidence, establishing a clear brain-behavior relationship. Comprehensive enhancements were noted in spatiotemporal gait parameters—including swing phase, step length, gait speed, and cadence—as well as in kinematic measures such as hip, knee, and ankle flexion angles and foot progression angle. In contrast, only limited improvements were seen in the single-target group (25, 26). These behavioral gains were underpinned by dual-target iTBS-induced neural reorganization. fNIRS analyses indicated that dual-target stimulation concurrently enhanced both global and local efficiency of brain networks and specifically strengthened functional connectivity between the affected M1 and unaffected prefrontal regions. This suggests that dual-target iTBS optimizes the balance between long-distance information transfer and local specialized processing—a cornerstone of efficient motor control (27, 28). In comparison, single-target iTBS only partially improved global integration without enhancing local connectivity. Furthermore, dual-target stimulation reversed the decrease in local connectivity within the affected M1 observed in both control and single-target groups, thereby mitigating maladaptive interhemispheric inhibition and promoting balanced bilateral network activation. Correlational analyses further substantiated this brain-behavior link, showing that improved step width in the dual-target group positively correlated with activation of the affected prefrontal cortex, highlighting the role of higher-order motor control mediated by this region in functional recovery. Previous iTBS studies on post-stroke lower limb rehabilitation have predominantly focused on single-target stimulation, such as the affected M1 or cerebellum (29, 30). For instance, Liao et al. (31) reported modest lower limb functional improvements with single-target M1 or cerebellar iTBS, though the effects were limited. A recent meta-analysis noted that the efficacy of single-target iTBS for lower limb recovery is constrained and exhibits considerable interindividual variability, likely due to its inability to modulate interconnected neural networks (32). These findings align with the present results. Our fNIRS data provide direct evidence that single-target iTBS not only failed to reverse the decline in local connectivity within the affected M1 but also induced negative correlations between gait improvements (e.g., swing phase, step width) and activation of the unaffected M1, possibly reflecting non-adaptive compensatory recruitment (9). By contrast, dual-target stimulation could activate cortex-cerebellum closed-loop circuit by co-activating the affected primary motor cortex and the contralateral cerebellum. Cerebellar iTBS is hypothesized to disinhibit the affected M1 via the dentato-thalamo-cortical pathway, thereby enhancing its excitability, while concurrent M1 stimulation further consolidates this effect (15, 33). This synergy ultimately manifests as strengthened functional connectivity between the affected M1 and ipsilateral PFC, alongside improved whole-brain network efficiency—representing a circuit-level optimization unattainable with single-target stimulation.

The findings have important clinical implications. Dual-target iTBS presents an innovative neuromodulation strategy for efficient and precise lower limb rehabilitation post-stroke. Clinicians should consider incorporating this cortex-cerebellum dual-target protocol into rehabilitation programs, particularly for patients with poor gait coordination and severe balance impairments. Moreover, our study identifies potential neural biomarkers for rehabilitation outcome assessment. The positive correlation between activation in the affected prefrontal cortex and gait improvement suggests that activation in this region may serve as a biological indicator of treatment response, potentially facilitating personalized rehabilitation.

This study has the following strengths: we integrated 3D gait analysis and fNIRS to synchronously correlate behavioral and neural changes, and employed a novel dual-target strategy targeting cortico-cerebellar circuits. However, some limitations need to be acknowledged: (1) This was a single-center study with a modest sample size (*n* = 63), potentially limiting the generalizability of our findings; (2) The study only assessed the immediate effect of the 21-day intervention, without any follow-up conducted after 1–6 months to evaluate the durability of the results. To address this limitation, follow-up visits at 3–6 months will be incorporated to verify the lasting impact of the intervention. Additionally, multiple time points will be included to determine the optimal duration of the intervention. (3) fNIRS did not perform in-depth assessment of the subcortical structures (e.g., cerebellar nuclei), which are critical for circuit function. Future studies are needed to address these gaps and explore synergies between central (cortico-cerebellar) and peripheral (e.g., spinal cord) stimulation, as suggested by recent research on “central-peripheral-central” neuromodulation (34).

## 5 Conclusion

Dual-target iTBS is superior to single-target iTBS and conventional rehabilitation for improving lower limb motor function, balance, and gait in stroke patients. This superiority is likely attributed to the activation of cortico-cerebellar circuits, which enhance M1-PFC connectivity, optimize brain network efficiency, and correct abnormal gait patterns. Correlation analyses further suggested that prefrontal activation on the affected side could be a potential neural marker to assess its efficacy. Clinically, these findings advocate for the use of dual-target iTBS to target cortico-cerebellar circuits, providing a promising neuromodulatory approach for post-stroke lower limb rehabilitation.

## Data Availability

The raw data supporting the conclusions of this article will be made available by the authors, without undue reservation.
