# Supplementary material for: Efficacy of dual-target iTBS on gait function and brain activation in stroke patients: a randomized, single-blinded, sham-controlled study
Source: Front Neurol. 2026 Jan 5;16:1678850. doi: 10.3389/fneur.2025.1678850 (PMC12812661; doi:10.3389/fneur.2025.1678850)
Supplement: Supplementary file 1 [file Data_Sheet_1.pdf]

3.4 Linear Regression Analysis Results

To further elucidate the neural mechanisms underlying the differential treatment effects of single-target and dual-target iTBS, we performed correlation analyses between post-treatment gait parameters and the mean activation values of key brain regions (as shown in the new Fig. 9). It is noteworthy that, for both groups, no other significant correlations were found between the remaining gait parameters and the other assessed brain regions (including the SMA and the contralesional PFC), with all p-values exceeding 0.05 (Supplementary Tables S1 and S2)

In the single-target (ST) group, the correlation analysis revealed a distinct pattern of negative associations. Specifically, we found that a longer swing phase and a narrower stride width were significantly correlated with lower activation levels in the primary motor cortex (M1) of the healthy hemisphere (H-M1) (Fig. 9A, B). This inverse relationship suggests that in patients receiving only M1 stimulation, better gait performance (evidenced by more normalized swing and step width) may be linked to a reduced reliance on compensatory mechanisms from the contralesional hemisphere. This finding aligns with the concept of reducing maladaptive cortical interference from the unaffected side as motor function recovers.

Supplementary Table S1. Complete correlation matrix between gait parameters and brain area means HbO values for the single-target (ST) group.

	Swing phase	Single support	Walking speed	Cadence	Step length	Stride width	Hip flexion	Knee fiexion	Ankle flexion	Foot deflection
H-M1										
r	-0.484	-0.089	-0.045	-0.241	0.152	-0.579	-0.203	-0.391	-0.029	0.004
P	0.026	0.702	0.847	0.292	0.512	0.006	0.377	0.080	0.901	0.987
A-M1										
r	-0.305	-0.052	0.015	-0.112	0.022	-0.372	-0.164	-0.128	0.244	0.017
P	0.179	0.823	0.947	0.630	0.923	0.096	0.478	0.580	0.286	0.942
H-SMA										

r	-0.358	-0.204	-0.210	-0.049	-0.133	-0.260	-0.285	-0.031	0.078	0.098
P	0.111	0.375	0.362	0.834	0.564	0.254	0.210	0.894	0.738	0.672
A-SMA										
r	-0.157	0.121	0.159	0.185	0.066	-0.314	-0.043	0.259	0.167	-0.195
P	0.496	0.602	0.490	0.421	0.776	0.166	0.853	0.258	0.470	0.396
H-PFC										
r	-0.043	0.130	0.245	0.248	0.284	-0.206	0.405	0.127	0.103	-0.314
P	0.854	0.513	0.284	0.275	0.212	0.371	0.068	0.584	0.658	0.165
A-PFC										
r	-0.292	-0.011	0.170	0.089	0.253	-0.181	0.132	0.043	0.028	-0.326
P	0.200	0.962	0.462	0.700	0.268	0.433	0.169	0.852	0.904	0.149

Abbreviations: H-, healthy side; A-, affected side

In contrast, the dual-target (DT) group exhibited a fundamentally different correlation profile. Here, functional improvements were associated with increased engagement of the affected hemisphere's prefrontal circuitry. We observed a significant positive correlation between step length and the mean HBO value in the prefrontal cortex (PFC) of the affected side (A-PFC) (Fig. 9C). This indicates that patients who achieved greater step length after dual-target stimulation also demonstrated higher levels of activation in the ipsilesional PFC. The PFC is critically involved in motor planning, executive control, and attention during complex tasks like walking. This positive correlation implies that the dual-target protocol may enhance gait function by successfully recruiting and integrating higher-order cognitive-motor resources in the damaged hemisphere, facilitating more purposeful and controlled locomotion.

Supplementary Table S2. Complete correlation matrix between gait parameters and brain area means HbO values for the dual-target (DT) group.

	Swing phase	Single support	Walking speed	Cadence	Step length	Stride width	Hip flexion	Knee fiexion	Ankle flexion	Foot deflection
H-M1										
r	-0.139	0.014	-0.118	0.284	0.010	0.303	-0.078	0.042	0.309	-0.164
P	0.547	0.434	0.882	0.213	0.967	0.183	0.736	0.856	0.173	0.036
A-M1										
r	0.117	0.180	0.035	0.190	-0.069	0.242	-0.359	-0.384	-0.008	-0.047
P	0.614	0.605	0.592	0.409	0.767	0.290	0.111	0.086	0.971	0.179
H-SMA										
r	-0.083	0.120	-0.124	-0.005	-0.180	0.305	-0.224	-0.359	-0.044	-0.006
P	0.719	0.667	0.203	0.984	0.436	0.179	0.330	0.110	0.581	0.070
A-SMA										
r	-0.197	-0.100	-0.290	-0.134	-0.127	0.133	-0.270	-0.234	0.156	0.016
P	0.392	0.833	0.268	0.563	0.583	0.566	0.237	0.307	0.501	0.501
H-PFC										
r	-0.107	-0.049	-0.255	-0.099	-0.274	0.243	-0.059	-0.088	-0.082	-0.154
P	0.644	0.096	0.265	0.670	0.229	0.288	0.799	0.703	0.723	0.052
A-PFC										
r	0.014	-0.373	-0.272	0.142	-0.191	0.441	-0.331	-0.285	0.126	-0.156
P	0.951	0.611	0.233	0.538	0.407	0.045	0.143	0.211	0.588	0.274

Abbreviations: H-, healthy side; A-, affected side
